# Aspergillus fumigatus Mitochondrial Acetyl Coenzyme A Acetyltransferase as an Antifungal Target

**DOI:** 10.1128/AEM.02986-19

**Published:** 2020-03-18

**Authors:** Yuanwei Zhang, Wenfan Wei, Jialu Fan, Cheng Jin, Ling Lu, Wenxia Fang

**Affiliations:** a Jiangsu Key Laboratory for Microbes and Functional Genomics, Jiangsu Engineering and Technology Research Centre for Microbiology, College of Life Sciences, Nanjing Normal University, Nanjing, China; b National Engineering Research Center for Non-Food Biorefinery, State Key Laboratory of Non-Food Biomass and Enzyme Technology, Guangxi Key Laboratory of Marine Natural Products and Combinatorial Biosynthesis Chemistry, Guangxi Academy of Sciences, Nanning, China; c School of Life Sciences, University of Science and Technology of China, Hefei, China; Michigan State University

**Keywords:** *Aspergillus fumigatus*, acetyl-CoA acetyltransferase, crystal structure, drug target, ergosterol biosynthesis

## Abstract

Ergosterol plays an important role in maintaining cell membrane sterol homeostasis in fungi, and as such, it is considered an effective target in antifungal chemotherapy. In yeast, the enzyme acetyl-coenzyme A (CoA) acetyltransferase (ERG10) catalyzes the Claisen condensation of two acetyl-CoA molecules to acetoacetyl-CoA in the ergosterol biosynthesis pathway and is reported as being critical for cell viability. Using yeast ERG10 for alignment, two orthologues, *Af*ERG10A (AFUB_000550) and *Af*ERG10B (AFUB_083570), were discovered in the opportunistic fungal pathogen Aspergillus fumigatus. Despite the essentiality of *Af*ERG10B having been previously validated, the biological function of *Af*ERG10A remains unclear. In this study, we have characterized recombinant *Af*ERG10A as a functional acetyl-CoA acetyltransferase catalyzing both synthetic and degradative reactions. Unexpectedly, *Af*ERG10A localizes to the mitochondria in A. fumigatus, as shown by C-terminal green fluorescent protein (GFP) tag fusion. Both knockout and inducible promoter strategies demonstrate that *Aferg10A* is essential for the survival of A. fumigatus. The reduced expression of *Aferg10A* leads to severe morphological defects and increased susceptibility to oxidative and cell wall stresses. Although the catalytic mechanism of acetyl-CoA acetyltransferase family is highly conserved, the crystal structure of *Af*ERG10A and its complex with CoA are solved, revealing four substitutions within the CoA binding site that are different from human orthologues. Taken together, our combination of genetic and structural studies demonstrates that mitochondrial *Af*ERG10A is essential for A. fumigatus cell viability and could be a potential drug target to feed the antifungal drug development pipeline.

**IMPORTANCE** A growing number of people worldwide are suffering from invasive aspergillosis caused by the human opportunistic fungal pathogen A. fumigatus. Current therapeutic options rely on a limited repertoire of antifungals. Ergosterol is an essential component of the fungal cell membrane as well as a target of current antifungals. Approximately 20 enzymes are involved in ergosterol biosynthesis, of which acetyl-CoA acetyltransferase (ACAT) is the first enzyme. Two ACATs in A. fumigatus are *Af*Erg10A and *Af*Erg10B. However, the biological function of *Af*Erg10A is yet to be investigated. In this study, we showed that *Af*Erg10A is localized in the mitochondria and is essential for A. fumigatus survival and morphological development. In combination with structural studies, we validated *Af*Erg10A as a potential drug target that will facilitate the development of novel antifungals and improve the efficiency of existing drugs.

## Abstract (precis)

A growing number of people worldwide are suffering from invasive aspergillosis caused by the human opportunistic fungal pathogen A. fumigatus. Current therapeutic options rely on a limited repertoire of antifungals. Ergosterol is an essential component of the fungal cell membrane as well as a target of current antifungals. Approximately 20 enzymes are involved in ergosterol biosynthesis, of which acetyl-CoA acetyltransferase (ACAT) is the first enzyme. Two ACATs in A. fumigatus are *Af*Erg10A and *Af*Erg10B. However, the biological function of *Af*Erg10A is yet to be investigated. In this study, we showed that *Af*Erg10A is localized in the mitochondria and is essential for A. fumigatus survival and morphological development. In combination with structural studies, we validated *Af*Erg10A as a potential drug target that will facilitate the development of novel antifungals and improve the efficiency of existing drugs.

## INTRODUCTION

The filamentous mold Aspergillus fumigatus is an opportunistic fungal pathogen ubiquitously found in nature. It causes a multitude of life-threatening diseases with high mortality rates in immunocompromised individuals, such as solid-organ transplant or bone marrow recipients and HIV-infected patients (1, 2). The azoles are considered the first-line antifungal agents in clinical treatment. However, over the past several years, numerous azole-resistant isolates of A. fumigatus have been found in many countries, including India, China, the United States, Australia, and the United Kingdom (3–6). Furthermore, drawbacks, such as side effects, toxicity, and/or emerging resistances (7) associated with other antifungal drug classes, make the development of new antifungal drugs imperative. The lack of chemically and genetically well-validated novel drug targets hinders antifungal discovery development (8). Crucial to the advancement of drug discovery pipeline is the identification and characterization of novel antifungal targets in A. fumigatus.

Fungal cell membranes possess ergosterol as the major lipid, which immensely contributes to membrane integrity, fluidity, and cell development (9). Ergosterol and enzymes involved in its biosynthesis have been considered attractive antifungal drug targets due to their uniqueness in fungi and absence in mammals (10). For instance, the widely used clinical antifungal amphotericin B targets ergosterol directly, whereas azole drugs target 14-α-sterol demethylase (CYP51), which is a key enzyme in the ergosterol pathway. The biosynthesis of ergosterol starts from acetyl-coenzyme A (acetyl-CoA) through a cascade of approximately 20 conserved enzymes in fungi (11). The first step is synthesized by acetyl-CoA acetyltransferase (ACAT) converting two acetyl-CoA into acetoacetyl-CoA, which is subsequently converted by 3-hydroxy-3-methyl-glutaryl (HMG)-CoA synthase and HMG-CoA reductase to mevalonate as the precursor for ergosterol biosynthesis (9). Mevalonate is involved not only in multiple cellular processes such as fungal growth, stress tolerance, and pathogenicity (12–14) but also as a key intermediate in the biosynthesis of siderophores under iron-limited conditions in A. fumigatus (15).

ACAT is the initial enzyme within the mevalonate and ergosterol biosynthesis pathway (16). It belongs to the thiolase superfamily, in which enzymes differ from each other in expression patterns, subcellular localizations, and substrate specificity (17). ACATs have been genetically characterized in many species. For instance, ACAT in Saccharomyces cerevisiae is encoded by the *erg10* gene and is demonstrated to be essential for growth (12). In Arabidopsis thaliana, ACAT1 and ACAT2 have been identified, and only ACAT2 is essential and required for normal growth and development (18). However, both are able to rescue the lethal phenotype of the S. cerevisiae *erg10* mutant, indicating that the function of ACAT is highly conserved (18). Similarly, Magnaporthe oryzae possesses two ACATs, *Mo*ACAT1 and *Mo*ACAT2. *Mo*ACAT2 is localized in cytoplasm and mainly contributes to vegetative growth and virulence, whereas a lack of mitochondrion-localized *Mo*ACAT1 did not trigger any growth defects (13). ACATs are also involved in regulating tumor cell metabolism (19, 20) and improving lipotoxicity and insulin resistance (21). In addition to genetic characterization, structures and kinetic properties of ACATs have been studied in Zoogloea ramigera, Clostridium acetobutylicum, S. cerevisiae, and humans (22–25). ACATs from these species form as tetramers composed of four identical monomers. Each monomer consists of three domains, the N-domain and the C-domain, which share the same βαβαβαββ topology, and the loop domain, which is involved in tetrameric assembly and provides binding pocket for the CoA moiety (22).

Validation of essential genes in fungi is crucial for expanding the target space and speeding antifungal drug development (26). As ACAT is the Achilles’ heel for mevalonate and ergosterol biosynthesis, we hypothesize that A. fumigatus ACAT is a potential antifungal target. In this study, by a combination of genetic characterization and structural investigation, we show that the mitochondrion-localized *Af*ERG10A in A. fumigatus is indispensable for viability. The displayed residue differences within the CoA binding site could be exploited for screening antifungal inhibitors or for the rational design of antifungal drugs.

## RESULTS

### *Aferg10A* (AFUB_000550) encodes an active acetyl-CoA acetyltransferase in A. fumigatus.

A BLASTp search of the A. fumigatus A1163 genome database using S. cerevisiae ERG10 (GenBank accession no. KZV07489.1) revealed two putative acetyl-CoA acetyltransferases, AFUB_000550 and AFUB_083570, referred to as *Af*ERG10A and *Af*ERG10B, respectively. Since *Af*ERG10B has been identified as being essential for the viability of A. fumigatus (27), *Af*ERG10A caught our interest. The *Aferg10A* gene is 1,424 bp in length, containing three exons and two introns, encoding *Af*ERG10A of 433 amino acids that shares 50% and 47% identity with human ACAT1 and ACAT2, respectively. To examine whether A. fumigatus *Af*ERG10A possesses the acetyl-CoA acetyltransferase activity, we overexpressed truncated *Af*ERG10A (residues 36 to 433) with a glutathione *S*-transferase (GST) fusion tag at the N terminus in Escherichia coli. After purification using glutathione beads, GST tag cleavage by PreScission protease, and gel filtration, 1 mg/liter pure *Af*ERG10A was obtained. It has been reported that acetyl-CoA acetyltransferase is capable of catalyzing both synthetic and degradative routes (24, 28, 29). Indeed, as shown in Table 1, *Af*ERG10A is active in both synthetic and degradative directions, with kinetic parameters comparable to those of orthologues from other species (22, 24, 30). The binding affinities for acetoacetyl-CoA and CoA are higher than that for acetyl-CoA (Table 1). Taken together, these data suggest that *Aferg10A* (AFUB_000550) encodes an active acetyl-CoA acetyltransferase in A. fumigatus.

**TABLE 1:** Michaelis-Menten kinetics parameters^*a*^

Protein	Degradative reaction with:				Synthetic reaction with acetyl-CoA	
	Acetoacetyl-CoA		CoA			
	*K*_m_ (μM)	*K*_cat_ (s^−1^)	*K*_m_ (μM)	*K*_cat_ (s^−1^)	*K*_m_ (μM)	*K*_cat_ (s^−1^)
*Af*ERG10A	43 ± 9	3 ± 0.2	26 ± 5	5 ± 0.2	232 ± 49	7 ± 0.4
H. sapiens ACAT1 (22)	4 ± 0.6	21 ± 1	20 ± 2	18 ± 2	508 ± 127	3.5 ± 0.7
*Z. ramigera* thiolase (30)	15	810	9	NA	1,200	71
E. coli ERG10 (24)	18	220	8	NA	139	7

a Data are presented as the mean with standard deviation of triplicates if available. NA, not available.

### *Af*ERG10A localizes to mitochondria in A. fumigatus.

**FIG 1:**
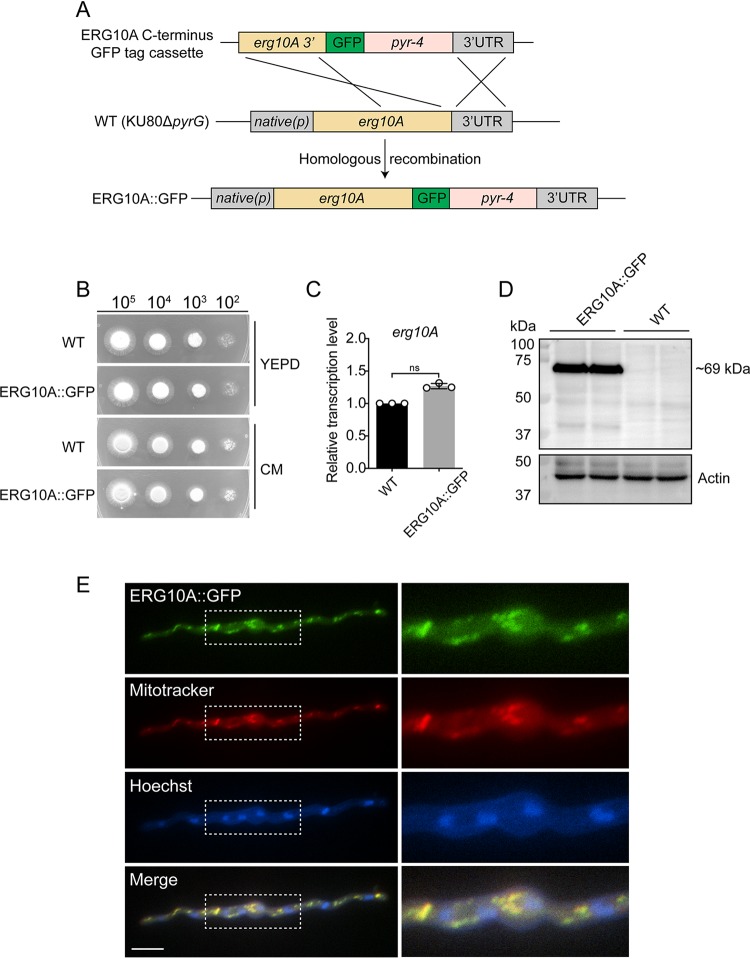
Subcellular localization of *Af*ERG10A. (A) Diagram illustrating the strategy for construction of the *Af*ERG10A::GFP strain under the control of a native promoter. (B) Colony morphologies of the indicated strains grown on YEPD medium and CM for 2 days at 37°C. (C) Expression analysis of the *erg10A* gene by qRT-PCR. Gene expression levels were normalized to the reference gene *tbp*. Error bars indicate the mean ± standard deviation (SD) of the results from three independent experiments. ns, not significant. (D) Western blot analysis of *Af*ERG10A::GFP strain. Actin was used as a loading control. The predicated size of the *Af*ERG10A-GFP fusion protein is 69 kDa. (E) Localization of *Af*ERG10A-GFP *in vivo*. Mitochondria and nuclei were stained by MitoTracker red and Hoechst stains, respectively. Scale bar = 10 μm.

As predicted by MitoProt (31), *Af*ERG10A has a mitochondrial targeting sequence at the N terminus, suggesting that it may localize to mitochondria (see Fig. S1 in the supplemental material). To evaluate this, a strain with a green fluorescent protein (GFP) tag fused at the C terminus of *Af*ERG10A was constructed (Fig. 1A). The growth of the *Af*ERG10A::GFP strain was not different from that of the wild type (WT) in both yeast extract-peptone-dextrose (YEPD) medium and complete medium (CM), and the mRNA expression level was not significantly different from that of the WT, indicating that the fusion *Af*ERG10A-GFP protein was fully functional (Fig. 1B and C). Western blot detection using GFP antibody revealed a specific band in *Af*ERG10A::GFP strain with molecular size of 69 kDa, approximately equivalent to the predicted size of the *Af*ERG10A-GFP fusion protein (42 + 27 kDa) (Fig. 1D). After 18 h of germination in minimal medium, the mitochondrion-specific fluorescent dye MitoTracker red and DNA dye Hoechst were used to stain the hyphae. The red fluorescence pattern well overlapped the GFP fluorescence signal (Fig. 1E), demonstrating that *Af*ERG10A localizes to mitochondria in A. fumigatus.

### *Af*ERG10A is essential for A. fumigatus survival.

**FIG 2:**
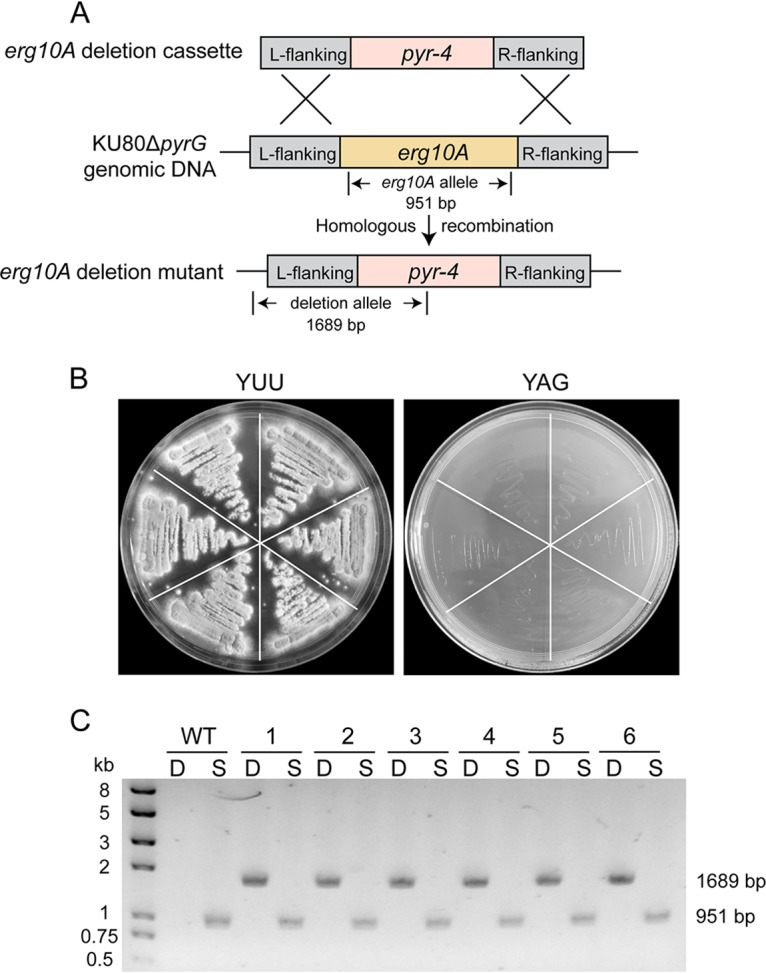
Heterokaryon rescue analysis of *Aferg10A* gene in A. fumigatus. (A) Diagram illustrating the deletion strategy for *Aferg10A*. (B) Conidia from six primary transformants by *Aferg10A* deletion cassette transformation were streaked on selective (YAG) and nonselective (YUU) plates and grown at 37°C for 48 h. (C) Diagnostic PCR showing that the WT only contains the *erg10A* gene allele (S, 951 bp, primers P27/P28) and that all six heterokaryons contain both that *erg10A* gene and deletion alleles (D, 1689 bp, primers P29/P30).

**FIG 3:**
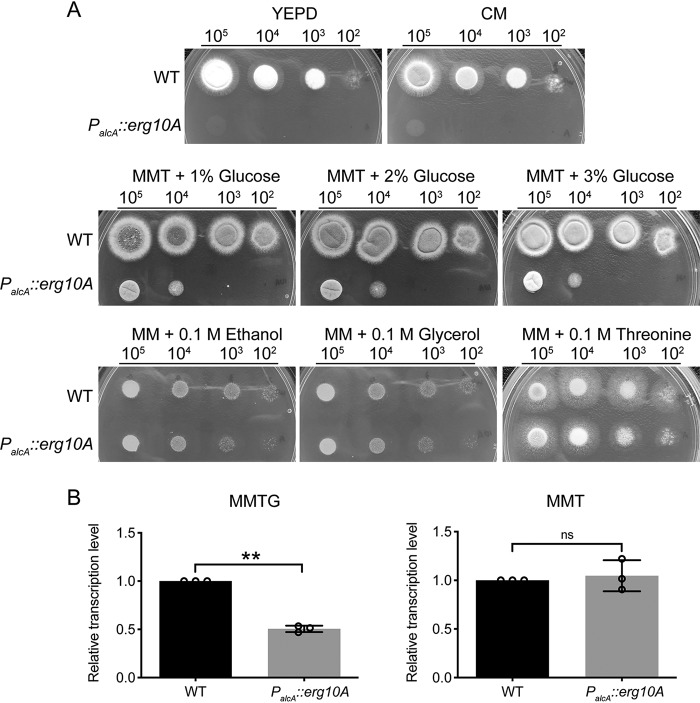
Growth phenotypes of the *P_alcA_*::*erg10A* conditional strain under inducing and repressing growth conditions. (A) Serial 10-fold dilutions of the indicated strains were inoculated on YEPD medium, CM, and MM supplemented with 0.1 M threonine, 0.1 M ethanol, 0.1 M glycerol, and 0.1 M threonine with 1 to 3% glucose for 2 days at 37°C. (B) qRT-PCR results of the mRNA expression level of the *Aferg10A* gene under induction (MMT) and partial-repression (MMTG) conditions. Gene expression levels were normalized to the reference gene *tbp*. Error bars indicate the mean ± SD of the results from three independent experiments. **, *P* < 0.001; ns, not significant.

To investigate the physiological role of *Aferg10A* in A. fumigatus, initially, we attempted to construct a null mutant by homologous recombination using the Neurospora crassa *pyr-4* selective marker to replace the *Aferg10A* gene (Fig. 2A). After several rounds of transformation and screening, no correct transformants were obtained, implying that *Aferg10A* might be essential for the viability of A. fumigatus. A heterokaryon rescue technique (32) was then applied to further dissect the essentiality of *Aferg10A*. The results showed that conidia from heterokaryons were not able to grow on selective medium (yeast extract glucose [YAG]) but grew well on nonselective medium (YAG supplemented with 5 mM uracil and 10 mM uridine) (Fig. 2B). Diagnostic PCR showed that the heterokaryons contained both the *Aferg10A* gene and the deletion alleles (Fig. 2C), confirming that *Aferg10A* is an essential gene in A. fumigatus like *Aferg10B* and the *erg10* gene in S. cerevisiae (12, 27). Alternatively, a conditional inactivation mutant was constructed by replacing the native promoter of the *Aferg10A* gene with an A. nidulans alcohol dehydrogenase promoter (*P_alcA_*), a tightly regulated promoter induced by ethanol, glycerol, or threonine but repressed by glucose and completely repressed on YEPD medium (33). After PCR analysis and Southern blotting, a correct conditional mutant was confirmed and referred to as *P_alcA_*::*erg10A* for phenotypic analysis (Fig. S2). Growth of the *P_alcA_*::*erg10A* strain was induced on solid minimal medium (MM) containing 0.1 M glycerol, 0.1 M ethanol, or 0.1 M threonine (MMT) as carbon sources. However, growth was completely inhibited on YEPD medium or CM and partially inhibited on MM containing 0.1 M threonine and 1% to 3% glucose, respectively (Fig. 3A), suggesting that the expression of *Aferg10A* is required for A. fumigatus viability. Quantitative real-time PCR was carried out to determine the transcriptional level of *Aferg10A* under induction conditions (MMT) and partial-repression conditions (MM with 0.1 M threonine and 1% glucose [MMTG]). The results showed that the mRNA level of *Aferg10A* in the *P_alcA_*::*erg10A* strain was comparable to that of the wild type (WT) in MMT but reduced to 50% of the WT when grown in MMTG (Fig. 3B). Since sufficient mycelia could be obtained from MMTG, this condition was selected for a subsequent experiment to analyze the physiological role of *Aferg10A*.

### Repression of *Aferg10A* results in severe growth defects in A. fumigatus.

**FIG 4:**
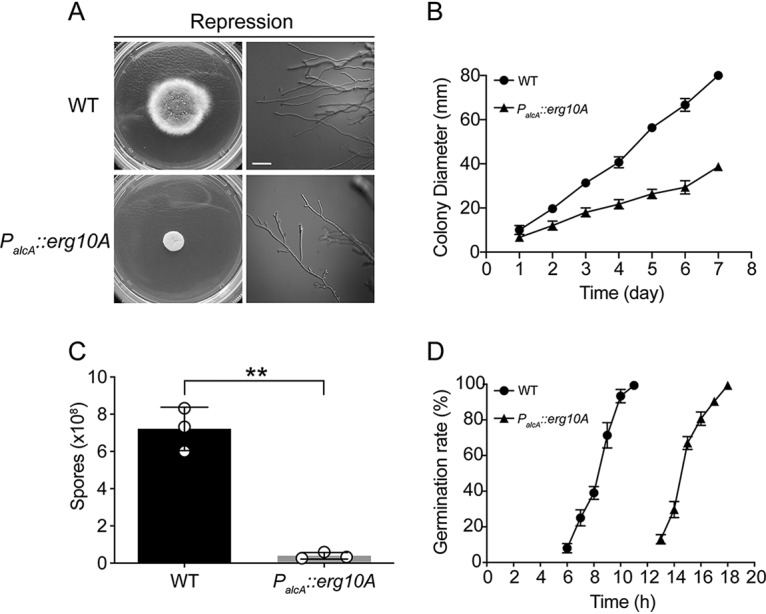
Morphological analysis of the *P_alcA_*::*erg10A* conditional mutant under partial-repressing conditions. (A) Colony and hyphal morphology of the indicated strains grown on partial-repression medium (MMTG) for 2 days at 37°C. Scale bar = 20 μm. (B) Conidia (1 × 10^6^) from the indicated strains were spotted onto MMTG at 37°C, and colony diameter was measured daily. Values represent the mean ± SD. (C) Quantitative data for the number of conidia of each strain grown on MMTG for 2 days at 37°C. Values represent the mean ± SD. **, *P* < 0.001. (D) Germination rate of the *P_alcA_*::*erg10A* conditional mutant and the WT. Conidia were incubated in stationary liquid MMTG at the time indicated. One hundred conidia for each strain were assessed for germination. These experiments were performed in triplicate. Values represent the mean ± SD.

The *P_alcA_*::*erg10A* conditional mutant and the WT were inoculated onto the solid MMTG plates to compare their morphologies. After 2 days of growth, the colony diameter of the *P_alcA_*::*erg10A* mutant was significantly smaller than that of the WT, and highly branched apical hyphae were seen in the mutant (Fig. 4A and B). In addition to hyphal growth, quantification of conidial production showed a 95% dramatic reduction in conidiation in the *P_alcA_*::*erg10A* mutant compared to the WT grown on MMTG for 48 h (Fig. 4C). Moreover, germination studies revealed that the germination initial time for the *P_alcA_*::*erg10A* mutant was 13 h, which was 7 h later than for the WT (Fig. 4D). Thus, these results demonstrated that the repression of *erg10A* results in severe growth defects in A. fumigatus.

### Repression of *Aferg10A* results in increased sensitivity to oxidative stresses and cell wall-perturbing agents.

**FIG 5:**
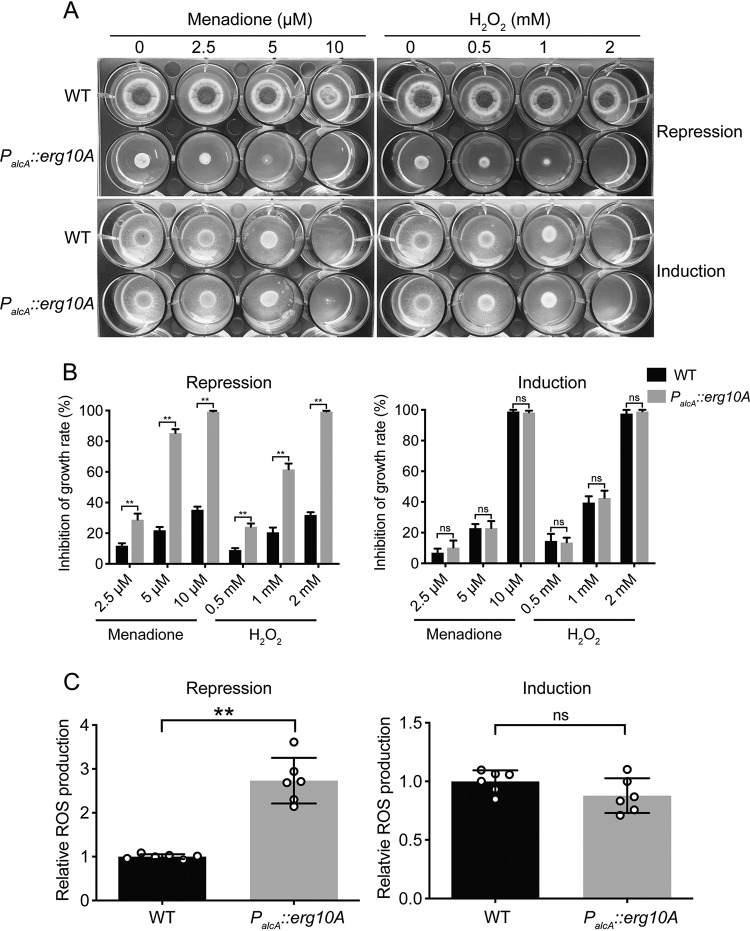
*Aferg10A* repression results in increased sensitivity to oxidative stresses. (A) Conidia (1 × 10^6^) of each indicated strain were inoculated on partial-repression (MMTG) or induction (MMT) medium containing a serial concentration of menadione and H_2_O_2_ for 2 days at 37°C. (B) Mycelium growth inhibition of the indicated strains on MMTG and MMT with different concentrations of menadione and H_2_O_2_. **, *P* < 0.001; ns, not significant. (C) ROS production of the *P_alcA_*::*erg10A* mutant and the WT under the partial-repressing (MMTG) and inducing (MMT) conditions. Data are presented as the means ± SD of the results from three biological replicates. Statistical signiﬁcance is indicated by *P* values: **, *P* < 0.001; ns, not significant.

**FIG 6:**
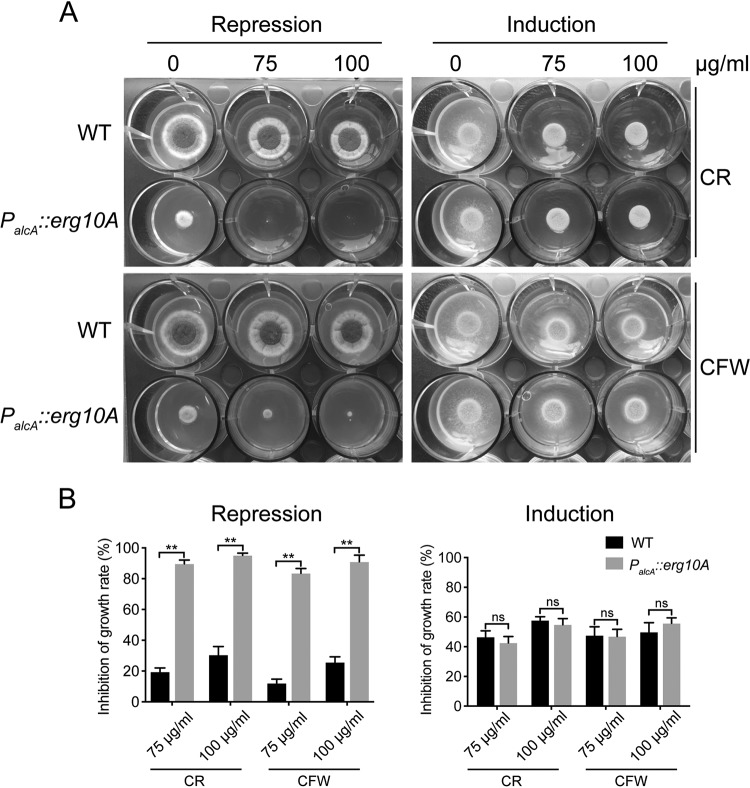
Repression of *Aferg10A* increases susceptibility to cell wall-perturbing agents. (A) Conidia (1 × 10^6^) of the indicated strains were inoculated onto 2 ml solid partial-repression (MMTG) or induction (MMT) medium in a 12-well plate supplemented with different concentrations of cell wall-perturbing agents, Congo red (CR) and calcofluor white (CFW), for 2 days at 37°C. (B) Mycelium growth inhibition of the indicated strains on MMTG and MMT with different concentrations of CR and CFW. **, *P* < 0.001; ns, not significant.

Previous studies have shown that mitochondrial function is associated with adaptation to oxidative stresses such as menadione and hydrogen peroxide treatments (34, 35); we therefore questioned whether mitochondrion-localized *Af*ERG10A is involved in adaptation to oxidative stresses or not. Under MMTG repression conditions, the *P_alcA_*::*erg10A* mutant showed an increased susceptibility to menadione and H_2_O_2_, whereas no such sensitivity was observed on induction MMT (induction medium) (Fig. 5A). Furthermore, the reactive oxidative stress (ROS) level was monitored using the oxidant-sensing probe 2′,7′-dichlorodihydrofluorescein diacetate (H2DCFDA), which is converted to highly fluorescent form by ROS. As shown in Fig. 5B, the ROS level in *P_alcA_*::*erg10A* mutant was significantly higher than in the WT (*P* < 0.001) in repression medium, whereas no difference was observed in inducing medium, suggesting that higher intercellular ROS level in *P_alcA_*::*erg10A* mutant may account for the increased sensitivity toward oxidative stresses. Previous study has shown that sterol C-24 reductase ERG4A/B is involved in cell wall integrity in A. fumigatus (36). Similarly, as shown in Fig. 6A and B, the *P_alcA_*::*erg10A* strain was hypersensitive to Congo red and calcofluor white under repression conditions but not induction conditions, suggesting that the repression of *Aferg10A* leads to defects in cell wall biosynthesis. Collectively, these results indicate that mitochondrion-localized *Af*ERG10A plays an important role in tolerance toward oxidative stress and cell wall stress in A. fumigatus.

### *Af*ERG10A crystal structure possesses exploitable differences in CoA binding site compared to human orthologues.

**FIG 7:**
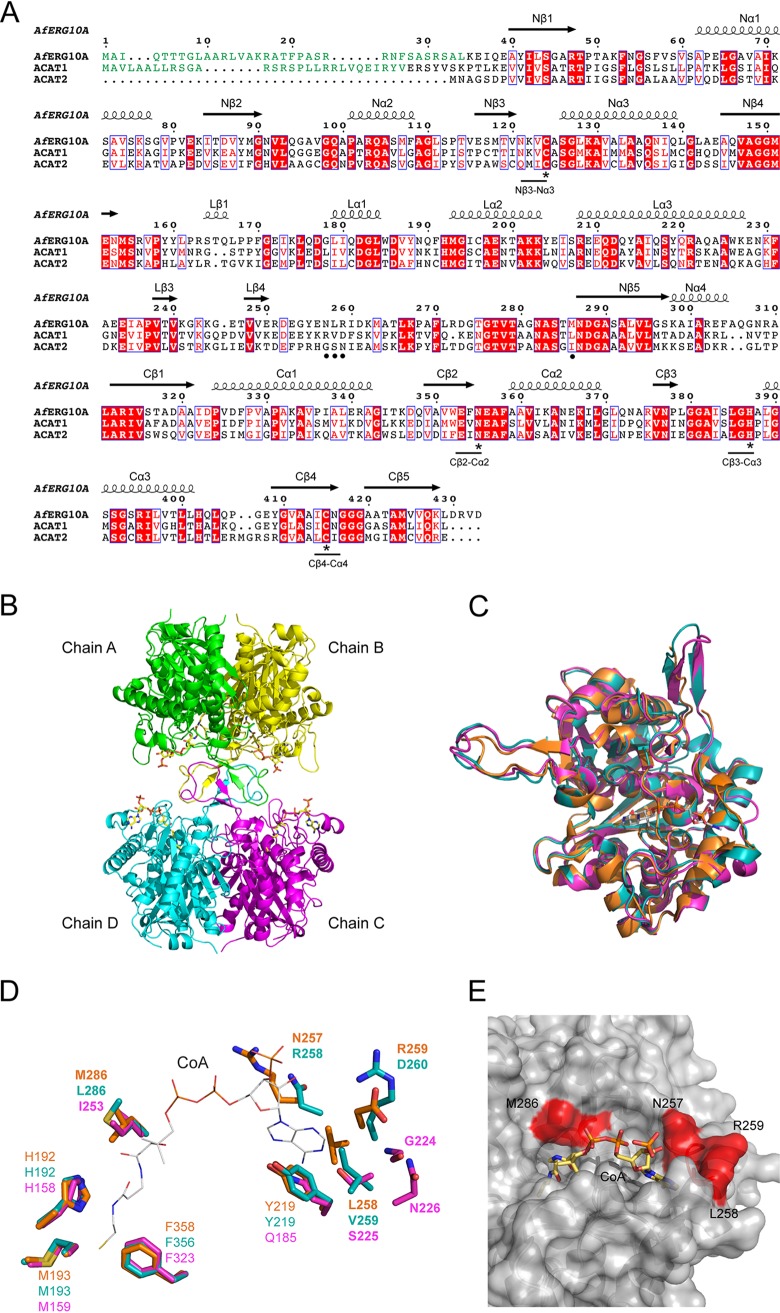
Sequence alignment and the crystal structure of *Af*ERG10A. (A) Clustal Omega (http://www.ebi.ac.uk/Tools/msa/clustalo) was used for the alignment of *Af*ERG10A with human ACAT1 and ACAT2. Mitochondrion-targeting sequences (MTS) of *Af*ERG10A and ACAT1 are colored in green. Highly conserved residues are shown in red letters and boxed; fully conserved residues are shown in red letters with red shading and boxed. The secondary structure of the *Af*ERG10A is indicated above the aligned sequences. Coils represent α helices, and arrows represent β strands; the N-terminal domain, C-terminal domain, and loop domain are labeled N, C, and L, respectively. The figure was prepared using the server ESPript3 (http://espript.ibcp.fr/ESPript/ESPript/) (58). Active sites are marked with asterisks, and four different residues within the CoA binding site compared to human orthologues are marked with dots. (B) Overall crystal structure of *Af*ERG10A tetramer in complex with CoA. The structure is shown as ribbons. Chains A to D are colored green, yellow, magenta, and cyan, respectively. CoA molecules are shown as sticks with yellow carbon atoms. (C) Superposition of the overall structure of *Af*ERG10A (orange), human ACAT1 (teal, PBD ID 2F2S), and ACAT2 (magenta, PDB ID 1WL5). CoA molecule is shown as sticks with gray carbon atoms. (D) Superposition of the CoA binding site residues in *Af*ERG10A with the corresponding residues in human ACAT1 (PBD ID 2F2S) and ACAT2 (PDB ID 1WL5). Carbon atoms of residues are shown as orange (*Af*ERG10A), teal (*Hs*ACAT1), and magenta (*Hs*ACAT2) sticks. The CoA molecule is shown as a thin line with gray carbon atoms. (E) Close-up view of the *Af*ERG10A CoA binding site. Conserved residues with *Hs*ACAT1 are colored in gray, and nonconserved substitutions are colored in red. The CoA molecule is shown as sticks with yellow carbon atoms.

Our genetic data here clearly demonstrate that *Af*ERG10A is essential for viability and could be a potential antifungal target against A. fumigatus. To provide insight into the structural prerequisites and find potential exploitable differences between *Af*ERG10A and human orthologues, structures of apo- and CoA-complexed *Af*ERG10A were determined using crystals grown from conditions of 0.1 M HEPES (pH 7.0) and 2 M ammonium sulfate in the absence or presence of CoA. Both structures were solved using human acetoacetyl-CoA thiolase (PDB identifier [ID] 2F2S) as the molecular model and refined to 2.4 Å with statistics as shown in Table S1. In one asymmetric unit, there are four molecules (A to D) forming a tetramer (Fig. 7B), which is similar to other members of thiolase family, such as in humans, S. cerevisiae, and E. coli (24, 25, 37). *Af*ERG10A consists of three domains, an N-terminal domain (residues Glu36 to Asn153 and Asn287 to Arg302), a C-terminal domain (residues Ala312 to Asp433), and a loop domain (residues Met154 to Met286) (Fig. 7A). By comparison, the root mean square deviation (RMSD) value between the apo- and CoA-bound structures is 0.175 Å, with 367 atoms matched, suggesting that CoA binding does not cause major conformation change.

The overall structure of *Af*ERG10A shares 49.6% sequence identity, with an RMSD of 0.51 Å on 313 Cα atoms to human mitochondrial ACAT1 (PDB ID 2F2S), as well as 44.9% sequence identity, with an RMSD of 0.57 Å on 313 Cα atoms to human cytosolic ACAT2 (PDB ID 1WL5) (Fig. 7A and C). The N-terminal and C-terminal halves have a βαβαβαββ topology and are connected by the loop domain of approximately 130 residues. The key active sites of ACAT1 and ACAT2 have been demonstrated to be Cys126, His385, and Cys413 in ACAT1 and Cys92, His353, and Cys383 in ACAT2 (24, 28). Sequence alignment of *Af*ERG10A with ACAT1 and ACAT2 revealed that the equivalent catalytic residues are Cys124, His387, and Cys415 in *Af*ERG10A (Fig. 7A). To confirm this, point mutations were introduced at the three sites as C124S, H387F, and C415S, and activity assays were conducted. As expected, the activity of these mutation variants could not be detected in either direction (data not shown), suggesting that these residues are essential for the catalytic activity of *Af*ERG10A.

Although the catalytic machinery of *Af*ERG10A and human ACATs is fully conserved, a close inspection of the CoA binding site revealed exploitable differences (Fig. 7D and E). For instance, residues binding to adenosine 3′-phosphate of CoA are Asn257, Leu258, and Arg259 in *Af*ERG10A, whereas they are Arg258, Val259, and Asp260 in ACAT1 and Gly225, Ser226, and Asn227 in ACAT2. Met286 in *Af*ERG10A binding to the pantetheine moieties of the CoA molecule is equivalent to Leu286 in ACAT1 and Ile254 in ACAT2. Those differences reveal that *Af*ERG10A possesses potentially exploitable differences in the CoA binding site compared to the human orthologues.

## DISCUSSION

As an important component of the fungal membrane, ergosterol is not only involved in numerous biological processes (10) but also associated with the host immune response by triggering macrophage pyroptosis (38). Thus, ergosterol and approximately 20 enzymes involved in its biosynthesis pathway are thought to be promising antifungal targets (39). Indeed, widely used antifungal drugs such as azoles, allylamines, and polyenes target ergosterol and its biosynthesis. Of the approximately 20 enzymes involved in ergosterol synthesis, only ERG3 (C-5 sterol desaturase), ERG4 (C-24 sterol reductase), ERG11 (lanosterol 14-α-demethylase), and ERG25 (C-4 methyl sterol oxidase) have been genetically characterized in the human opportunistic pathogen A. fumigatus (27, 36, 40, 41). The physiological functions of the other enzymes involved in ergosterol biosynthesis are yet to be explored, including acetyl-CoA acetyltransferase ERG10, which is the initial enzyme in the mevalonate and ergosterol biosynthesis pathway.

Redundancy has been reported in several ergosterol synthesis enzymes in A. fumigatus. For example, ERG4, ERG11, and ERG25 are encoded by two gene copies, whereas ERG3 is encoded by three gene copies (10). Not surprisingly, phylogenetic analysis revealed two copies of ERG10 in A. fumigatus, *Af*ERG10A and *Af*ERG10B (Fig. S1). *Af*ERG10B has been validated as essential (27) and similar to those homologues which do not have a mitochondrial targeting sequence (MTS) (Fig. S1), whereas *Af*ERG10A contains a predicted MTS similar to human ACAT1 and ERG10 from *M. oryzae* and Caenorhabditis elegans. By constructing a C-terminal GFP-tagged *Af*ERG10A::GFP strain, indeed, we verified that *Af*ERG10A is localized to the mitochondria (Fig. 1). Enzymatic characterization of recombinant *Af*ERG10A demonstrated that it is capable of catalyzing both synthetic and degradative reactions. The *K_m_* of *Af*ERG10A for acetoacetyl-CoA and CoA was lower than that for acetyl-CoA, suggesting that *Af*ERG10A may prefer a degradative reaction (Table 1), which is similar to its orthologue in E. coli (24).

By heterokaryon rescue and growth of the conditional mutant under repression conditions, we confirmed the essentiality of *Af*ERG10A (Fig. 2 and 3). Interestingly, a previous study using a nitrogen-regulated NiiA promoter (*P_NiiA_*) replacement to identify essential genes in A. fumigatus demonstrated that *Af*ERG10B is also essential for cell viability (27). It seems like neither *Af*ERG10A nor *Af*ERG10B could functionally compensate for each other. This is further validated when only slightly increased expression of *Aferg10B* (approximately 1.8-fold) was detected under partial repression of *Aferg10A* (Fig. S3A). However, constitutive expression of S. cerevisiae *erg10* in the *P_alcA_*::*erg10A* strain fully restored the growth of the mutant under both complete and partial-repression conditions (Fig. S3C), suggesting a conserved function between yeast ERG10 and *Af*ERG10A. It is reasonable to propose that *Af*ERG10A and *Af*ERG10B execute different physiological functions strictly due to spatial localization, but both are indispensable for viability in A. fumigatus. In contrast, the deletion of either cytoplasmic or mitochondrial acetyl-CoA acetyltransferases in *M. oryzae* was not lethal (13), implying different biological roles of ACATs in filamentous fungi.

The reduced expression of *Aferg10A* resulted in a significant loss of radial growth, retarded germination, and hyperbranching, revealing the important role of *Af*ERG10A in growth and morphogenesis (Fig. 4). The repression of *Aferg10A* also led to increased sensitivity to oxidative stresses and a high ROS level (Fig. 5), providing evidence that mitochondrion-localized *Af*ERG10A plays an important role in maintaining mitochondrial function. Moreover, the *P_alcA_*::*erg10A* conditional mutant displayed increased sensitivity toward cell wall-perturbing agents under partial-repression conditions (Fig. 6), consistent with previous studies showing that ERG4 and ERG5 (C-22 sterol desaturase) in ergosterol biosynthesis are required for cell wall assembly (36, 42). Surprisingly, the conditional mutant did not show susceptibility to clinical polyene and azole drugs, which target ergosterol and its biosynthesis enzyme, respectively (Fig. S3D), suggesting that the repression of *Aferg10A* has no effect on ergosterol synthesis. Based on the kinetics parameters and the nonsusceptible feature of the *P_alcA_*::*erg10A* mutant toward azole and polyene drugs, we propose that the degradative reaction catalyzed by *Af*ERG10A may be important for acetyl-CoA homeostasis in mitochondrial metabolic pathways such as the citric acid cycle.

To date, only a single inhibitor, arecoline hydrobromide (AH), against the thiolase family was identified by *in vitro* screening in cancer cells. AH disrupted the transformation between tetrameric and monomeric of ACAT1 which is critical for cancer cell proliferation (20). The essentiality and pleiotropic function of *Af*ERG10A indicate that it could be a potential antifungal drug target. However, due to the high sequence conservation between *Af*ERG10A and human ACATs, compounds targeting *Af*ERG10A are prone to causing toxicity effects. Encouragingly, by solving the crystal structure of *Af*ERG10A and superimposing it with structures of human orthologues, four different residues within the CoA binding site were discovered (Fig. 7C and D). This will be the structural basis for a rational design of specific antifungal inhibitors targeting *Af*ERG10A. Recently, fragment-based drug discovery has been successfully applied to explore selective inhibitors against highly conserved potent targets in both academia research and pharmaceutical companies (43). The small fragments used in this approach have the possibility of binding to the nonconserved site of the target. Then, the selective inhibitor can be obtained by iterative cycles of chemical optimization guided by structural information with an *in vitro* enzymatic or *in vivo* inhibition assay. For example, selective inhibitors have been successfully identified and optimized based on the only single residue difference between Plasmodium falciparum *N*-myristoyltransferase (NMT) and a human orthologue (44, 45).

In summary, here, we biochemically, genetically, and structurally characterized mitochondrion-localized *Af*ERG10A in A. fumigatus as an attractive drug target to feed the current antifungal drug development pipeline.

## MATERIALS AND METHODS

### Strains and culture conditions.

The A. fumigatus KU80 Δ*pyrG* strain was used as the recipient for the generation of GFP labeling and mutant strains, whereas KU80 was used as the wild type (WT) for functional analysis (46). Strains were cultured on minimal medium (MM), YEPD medium, and complete medium (CM) containing different supplements, as described previously (47, 48). Mycelia from liquid medium at 37°C with shaking at 200 rpm for a speciﬁed culture time point were harvested, washed with distilled water, frozen in liquid nitrogen, and then ground using a mortar and pestle. The mycelium powder was stored at −80°C for DNA, RNA, and protein extraction. The spores were collected by using 0.02% (vol/vol) Tween 20 in a saline solution from plates with 48 h of incubation at 37°C.

### Construction of the A. fumigatus *erg10A* mutants.

To generate a conditional inactivation mutant, plasmid pAL3 (33) containing the Aspergillus nidulans alcohol dehydrogenase promoter (*P_alcA_*) and the N. crassa *pyr-4* gene was used to construct a suitable vector allowing the replacement of the native promoter of the *Aferg10A* by *P_alcA_*. A fragment of 1,116 bp from −60 to +1056 of the *Aferg10A* genomic DNA sequence was amplified with primers P7 and P8. The PCR-amplified fragment was digested with SmaI and XbaI and then subcloned into the vector pAL3 to yield pALERG10N and confirmed by sequencing. pALERG10N was used to transform KU80 Δ*pyrG* protoplasts by polyethylene glycol (PEG)-mediated fusion (49), and positive transformants were selected by uridine/uracil autotrophy on MM with threonine as the sole carbon source. The transformants were confirmed by PCR using three pairs of primers (P1/P2, P3/P4, and P5/P6) (Table 2). Primers P1 and P2 were used to amplify a 1,424-bp fragment of the *Aferg10A* gene. P3 and P4 were used to amplify a 1,852-bp fragment from *P_alcA_* to a downstream flanking region of the *Aferg10A* gene. Primers P5 and P6 were used to amplify the N. crassa *pyr-4* gene. To generate the C-terminal GFP-tagged *Af*ERG10A::GFP strain under the control of a native promoter, the 1,421-bp gene coding region without a stop codon and the 1,503-bp downstream sequence starting from the stop codon were ampliﬁed using P21/P22 and P23/P24, respectively. Next, we cloned the two fragments into vector pFNO3-GFP (50) containing a GFP tag and *pyrG* marker and then transformed them into the KU80 Δ*pyrG* protoplast. Transformants were selected by uridine/uracil autotrophy on minimal medium. For complementation of the *Aferg10A* conditional mutant with the S. cerevisiae *erg10* gene, the *P_alcA_*::*erg10A* strain was subjected to 1 mg/ml 5-fluorotic acid (5-FOA) to inactivate the *pyr-4* marker, obtaining the *P_alcA_*::*erg10A pyr-4* auxotrophic strain. The open reading frame (ORF) of *Scerg10* was amplified from the S. cerevisiae S288c genomic DNA with primers P25/P26 and subcloned into the vector pGPDUU, which contains a glyceraldehyde 3-phosphate dehydrogenase (GPD) constitutive promoter and *pyr-4* marker. The resulting plasmid was used to transform *P_alcA_*::*erg10A pyr-4* auxotrophic protoplasts. For Southern blot analysis, 10 μg of genomic DNA of the WT and *erg10A* conditional strains was digested with XbaI. Labeling and visualization were performed using the digoxigenin (DIG) DNA labeling and detection kit (Roche Applied Science), according to the manufacturer’s instructions. The heterokaryon rescue technique was performed as described previously (32).

**TABLE 2:** Primers used in this study

Name	Sequence (5′–3′)
P1	ATGGCGATTCAAACAACAAC
P2	TCAGTCGACCCGATCAA
P3	CCGACCTAGGATTGGATGCA
P4	GGTATAATCATGGCGTGTCGC
P5	AAACGCAAATCACAACAGCCAAC
P6	CTATGCCAGACGCTCCCGG
P7	GTACCCGGGTTCTGAAGCTCTCTCATATTCACCTTATAG
P8	TCCTCTAGAGGCAGGGGCAACGGGGAAGTCCACTG
P9	GGAGGCTTACATTCTCAGTGG
P10	TTCTCAACAGGAACGCCAG
P11	CCACCTTGCAAAACATTGTT
P12	TACTCTGCATTTCGCGCATG
P13	AAAGGATCCAAAGAGATTCAGGAGGCTTACATTCTCAG
P14	AAAGCGGCCGCTCAGTCGACCCGATCAAGCTTCTGAAC
P15	CATGACGGTAAACAAAGTGAGCGCATCTGGCCTCAAAG
P16	CTTTGAGGCCAGATGCGCTCACTTTGTTTACCGTCATG
P17	GAGCCATTTCCCTGGGATTCGCCCTGGGAAGCTCTG
P18	CAGAGCTTCCCAGGGCGAATCCCAGGGAAATGGCTC
P19	GTGTGGCTGCAATCAGCAACGGCGGTGGTGCTG
P20	CAGCACCACCGCCGTTGCTGATTGCAGCCACAC
P21	CATCACCGAATTCTGGCAATGTCTAGAATGGCGATTCAAACAACAACTGGGTTG
P22	GCTCCAGCGCCTGCACCAGCTCCGTCGACCCGATCAAGCTTCTGAACAAC
P23	CGCATCAGTGCCTCCTCTCAGACAGTGAGCCACTGGCCGTTGATTAACGTTC
P24	CGGAGAGAGATTCTTCTGCTGTACTAGTGCGGTAGACGCGTTCGCCAGCATC
P25	GGTGCAGGCGCTGGAGCCGGTGCCATGTCTCAGAACGTTTACAT
P26	GAGCATTGTTTGAGGCGACCGGTTTATCATATCTTTTCAATGACAA
P27	ACCCCTTGCCGTCTTCTTTA
P28	CATTGAACTCCCAAACCGCA
P29	CCACCGGCTTGAGAGAATTG
P30	GGTCCAAGTGGAAGTAGGTAGTGAC
P31	GAAGAGATTGCCCCTATCCAG
P32	GTCCCAGAACCAGGTATAAACG

### Quantitative real-time PCR.

Quantitative real-time PCR (qRT-PCR) was performed with PerfeCta SYBR green FastMix (Quanta BioSciences) using a Rotor-Gene Q real-time PCR system (Qiagen). Total RNAs from the spores cultured in liquid MM supplemented with 0.1 M threonine (MMT) or 1% glucose (MMTG) at 37°C and 200 rpm for 48 h were extracted using the TRIzol reagent (Invitrogen). cDNA synthesis was performed with 1.5 μg of RNA using the qScript cDNA SuperMix (Quanta BioSciences), according to the manufacturer’s instructions. Primers P9 and P10 were used to amplify a fragment of *Aferg10A*, primers P31 and P32 were used to amplify a fragment of *Aferg10B*, and primers P11 and P12 were used to amplify the 80-bp *tbp* gene, encoding the TATA box-binding protein, as the reference gene. The thermal cycling conditions were 95°C for 2 min, followed by 45 cycles of 95°C for 15 s and 60°C for 60 s. Real-time PCR data were acquired using Sequence Detection software. The standard curve method was used to analyze the real-time PCR data. Samples isolated from different strains at different times were tested in triplicate.

### Analysis of the *P_alcA_*::*erg10A* conditional mutant.

To test sensitivity toward cell wall and oxidative stresses, 10^6^ conidia of the *P_alcA_*::*erg10A* conditional mutant and the WT were spotted on MMT and MMTG plates containing various concentrations of calcofluor white, Congo red, menadione, and H_2_O_2_. After incubation at 37°C for 48 h, the plates were taken out and photographed.

For the conidial germination assay, strains were grown in liquid MMTG with an inoculum of 10^6^ conidia/ml for indicated time points. Germination rate was determined by counting a total of 100 spores and recording the number of germinated spores. Counting was repeated three times for each strain, and the mean and standard deviations were calculated.

Radial growth rate was determined by spotting 10^4^ conidia onto the center of a plate and monitoring colony diameter daily. After 2 days of incubation, spores were harvested and counted to compare sporulation differences.

### Western blot analysis.

Western blotting was carried out as previously reported (51). Briefly, 1 × 10^6^ conidia of the *Af*ERG10A::GFP and WT strains were inoculated in liquid CM and shaken at 37°C for 48 h. The mycelia were harvested and ground in liquid nitrogen with a mortar and pestle and suspended in ice-cold extraction buffer (50 mM HEPES [pH 7.4], 137 mM KCl, 10% glycerol, 1 mM EDTA, 1 μg/ml pepstatin A, 1 μg/ml leupeptin, 1 mM phenylmethylsulfonyl fluoride [PMSF]). Ten micrograms of proteins per lane was loaded onto a 10% SDS-PAGE gel. After electrophoresis, proteins were transferred to a polyvinylidene difluoride (PVDF) membrane (Millipore) in 384 mM glycine, 50 mM Tris (pH 8.4), and 20% methanol at 250 mA for 1.5 h. The membrane was then blocked with phosphate-buffered saline (PBS) containing 5% milk and 0.1% Tween 20. The membrane was then incubated in anti-mouse GFP primary antibody (Roche) at a 1:20,000 dilution and goat anti-mouse IgG-horseradish peroxidase secondary antibody at a 1:5,000 dilution. Blots were developed using the Clarity ECL Western blotting detection reagents (Bio-Rad), and images were acquired with a Tanon 4200 chemiluminescent imaging system (Tanon).

### Fluorescence microscopy.

To visualize the localization of ERG10A-GFP, 1 × 10^6^ conidia of the *Af*ERG10A::GFP strain were incubated on glass coverslips in 2 ml MM at 37°C for 10 h. Samples were incubated with 25 nM MitoTracker red CMXRos (Invitrogen) for 5 min at room temperature and washed three times with PBS. Then, the cells were fixed for 20 min in 4% (vol/vol) formaldehyde at room temperature, and nuclei were stained with Hoechst solution at a final concentration of 0.1 mg/ml. All images were captured using the Axio imager A1 fluorescence microscope (Carl Zeiss, Jena, Germany).

### Measurement of reactive oxygen species.

The production of reactive oxygen species was measured as described previously (50, 52), with minor changes. Brieﬂy, 10^7^ spores were incubated in 100 ml medium at 37°C for 24 h with shaking at 220 rpm. Then, 30 μM 2′,7′-dichlorodihydroﬂuorescein diacetate (H2DCFDA; Invitrogen) was added to the medium and incubated at 37°C for 1.5 h. After this, the mycelia were harvested and washed three times with distilled water to remove extracellular H2DCFDA. The ﬁltered mycelia were then ground in liquid nitrogen and suspended in PBS. The resulting supernatant was collected by centrifugation at 15,000 × *g* and 4°C for 10 min. Fluorescence was measured using a SpectraMax M2 reader (Molecular Devices, USA), with an excitation wavelength of 504 nm and an emission wavelength of 524 nm. The ﬂuorescence intensity was normalized to the protein concentration of the sample, which was measured using a Thermo Coomassie protein assay kit.

### Cloning of A. fumigatus ERG10A.

The *Aferg10A* gene was amplified from cDNA by PCR using the forward primer P13 and the reverse primer P14, and it was then cloned into the pGEX-6P1 vector (GE Healthcare) containing a glutathione *S*-transferase (GST) tag, followed by a PreScission protease cleavage site, yielding the expression plasmid pGEX-*Af*ERG10A_36–433_. The variants C124S, H387F, and C415S were created using pGEX-*Af*ERG10A_36–433_ as the template with primers P15/P16, P17/P18, and P19/P20 using the QuikChange site-directed mutagenesis kit (Stratagene). All plasmids were verified by a sequencing service.

### Expression and purification of *Af*ERG10A.

*Af*ERG10A and the C124S, H387F, and C415S mutated forms were expressed and purified as described previously (53). Briefly, the N-terminally truncated pGEX-*Af*ERG10A_36–433_ and mutated forms were transformed into E. coli BL21(DE3)/pLysS and a single colony inoculated into 100 ml of Luria-Bertani (LB) medium containing 0.1 mg/ml ampicillin and incubated at 37°C for 18 h with shaking at 200 rpm. Ten milliliters of the culture was used to inoculate 1 liter LB medium and grown to an optical density at 600 nm (OD_600_) of 0.6. Expression of the GST fusion protein was induced by 250 μM isopropyl-β-d-thiogalactopyranoside (IPTG) at 16°C for a further 20 h. The cells were then harvested by centrifugation at 4,000 × *g* for 30 min, resuspended in 25 ml lysis buffer (25 mM HEPES, 150 mM NaCl [pH 7.5] containing 10 mg/ml DNase, 0.5 mg/ml lysozyme, and a tablet of protease inhibitor cocktail [Roche]), and lysed using a French press at 650 lb/in^2^. The cell lysate was centrifuged at 20,000 × *g* for 30 min to remove cell debris, and the supernatant was incubated with prewashed glutathione Sepharose beads (GE Healthcare) at 4°C on a rotating platform for 2 h. The GST-*Af*ERG10A fusion protein was isolated by centrifugation and washed with buffer (25 mM HEPES, 150 mM NaCl [pH 7.5]). The GST tag was cleaved by PreScission protease (50 μg protease per ml of beads) for 18 h at 4°C. The cleaved protein was filtered from the beads, concentrated to 5 ml using a 10-kDa cutoff Vivaspin concentrator (GE Healthcare), loaded onto a Superdex 200 column (Amersham Bioscience) equilibrated with buffer (25 mM HEPES, 150 mM NaCl [pH 7.5]), and eluted at a flow rate of 1 ml/min in the same buffer. The fractions were verified by SDS-PAGE. Pure fractions were pooled and concentrated to 16 mg/ml and stored at −80°C for later use.

### Steady-state kinetics.

*Af*ERG10A activity was determined using Mg^2+^ method, as reported previously (28, 37). In the degradative reaction, a 100-μl reaction mixture containing 50 mM Tris-HCl (pH 8.1), 20 mM MgCl_2_, 40 mM KCl, 60 μM CoA, and 10 μM acetoacetyl-CoA was incubated at 30°C for 5 min. For determining the *K_m_* for acetoacetyl-CoA, concentrations of 10, 25, 50, 75, 100, 150, and 200 μM acetoacetyl-CoA were applied. For determination of the *K_m_* for CoA, concentrations of 10, 20, 40, 60, 80, 100, and 120 μM CoA were used. In the synthetic reaction, a 100-μl reaction mixture consisting of 50 mM Tris-HCl (pH 7.4), 0.2 mM NADH, 1 unit of l-3-hydroxyacyl-CoA dehydrogenase, 0.5 mM dithiothreitol (DTT), and various concentrations (100, 200, 400, 800, 1,000, and 1,200 μM) of acetyl-CoA was incubated at 30°C for 5 min. The reduction rate of NADH was determined at 340 nm by a SpectraMax i3x reader (Molecular Devices). An extinction coefficient of 6,220 M^−1 ^cm^−1^ was used for activity calculation.

### Crystallization, data collection, and structure determination.

Crystallizations were carried out at room temperature using the sitting drop method. Each drop contained an equal volume of 0.2 μl of protein (10 mg/ml) and 0.2 μl of reservoir solution. Crystals appeared under conditions of 0.1 M HEPES (pH 7.0) and 2 M ammonium sulfate. A single crystal was cryoprotected by 20% glycerol and sent for diffraction at the European Synchrotron Radiation Facility (ESRF; Grenoble, France) on beamline ID23-1, and data were processed using the HKL suite (54). The structure was solved by MOLREP using the human mitochondrial acetyl-CoA acetyltransferase ACAT1 structure (PDB ID 2F2S) as the search model. REFMAC (55) was used for further refinement, and Coot was used for iterated model building (56). Figures were produced by PyMOL (57).

### Data availability.

The atomic coordinates and structure factors of *Af*ERG10A and the *Af*ERG10A-CoA complex were deposited in the Protein Data Bank with accession codes 6L2G and 6L2C.

## Supplementary Material

Supplemental file 1
