# Supplementary material for: Aspergillus fumigatus Mitochondrial Acetyl Coenzyme A Acetyltransferase as an Antifungal Target
Source: Appl Environ Microbiol. 2020 Mar 18;86(7):e02986-19. doi: 10.1128/AEM.02986-19 (PMC7082573; doi:10.1128/AEM.02986-19)
Supplement: Supplemental file 1 [file AEM.02986-19-s0001.pdf]

Characterization of *Aspergillus fumigatus* mitochondrial acetyl-CoA acetyltransferase as an antifungal target

Yuanwei Zhang[a], Wenfan Wei[c], Jialu Fan[a], Cheng Jin[b], Ling Lu[a] and Wenxia Fang[b#]

[a]Jiangsu Key Laboratory for Microbes and Functional Genomics, Jiangsu Engineering and Technology Research Centre for Microbiology, College of Life Sciences, Nanjing Normal University, Nanjing, China

[b]National Engineering Research Center for Non-Food Biorefinery, State Key Laboratory of Non-Food Biomass and Enzyme Technology, Guangxi Key Laboratory of Marine Natural Products and Combinatorial Biosynthesis Chemistry, Guangxi Academy of Sciences, Nanning, China

[c]School of Life Sciences, University of Science and Technology of China, Hefei, China

Running title: *Af*ERG10A as an antifungal target

[#]Address correspondence to Wenxia Fang, wfang@gxas.cn

Supplementary figures

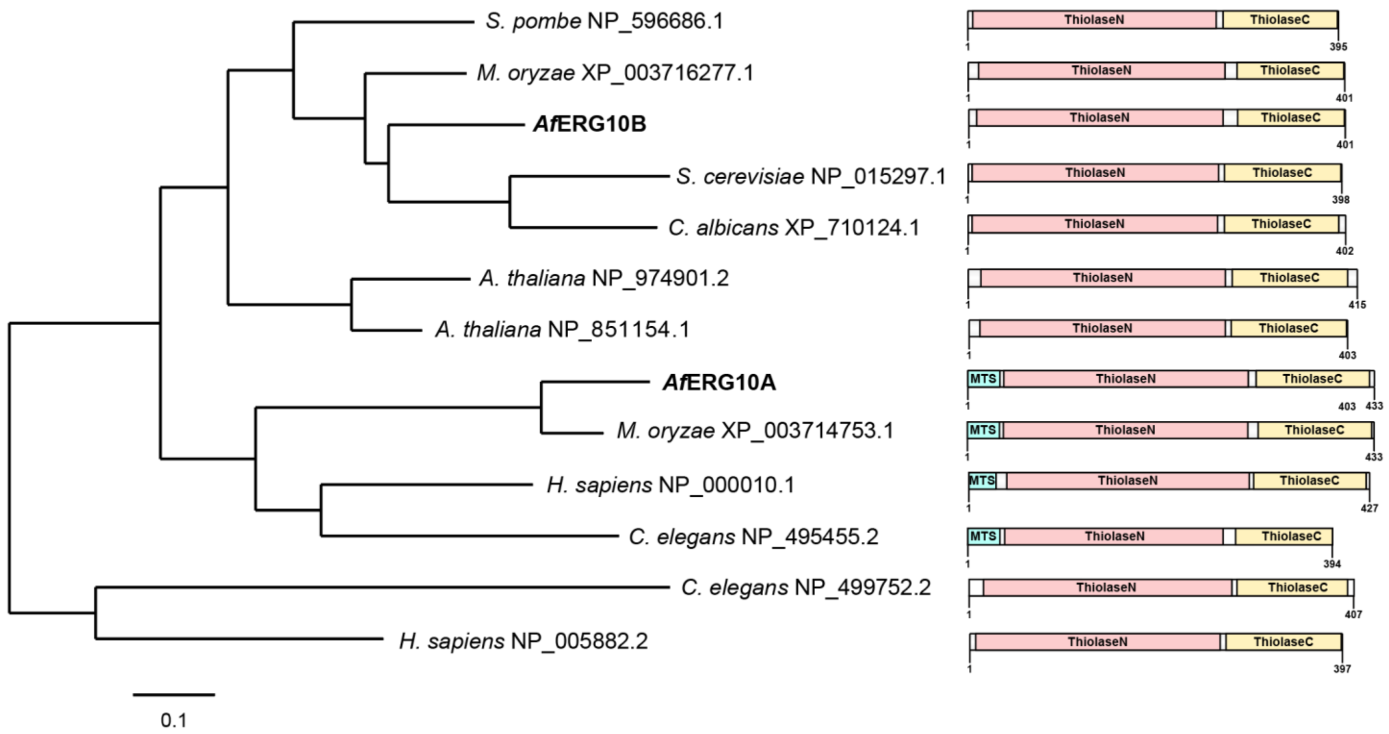

Figure S1. Phylogenetic analysis of *Af*ERG10A homologs in other species.

Phylogenetic analysis was carried out at http://www.phylogeny.fr/index.cgi using the maximum likelihood method. Species names are shown in the figure followed by the GenBank accession number. The predicted domain was assessed using the SMART interface (http://smart.embl-heidelberg.de/). Domain architectures of each protein are drawn by IBS software (1). MTS: mitochondrial targeting sequence.

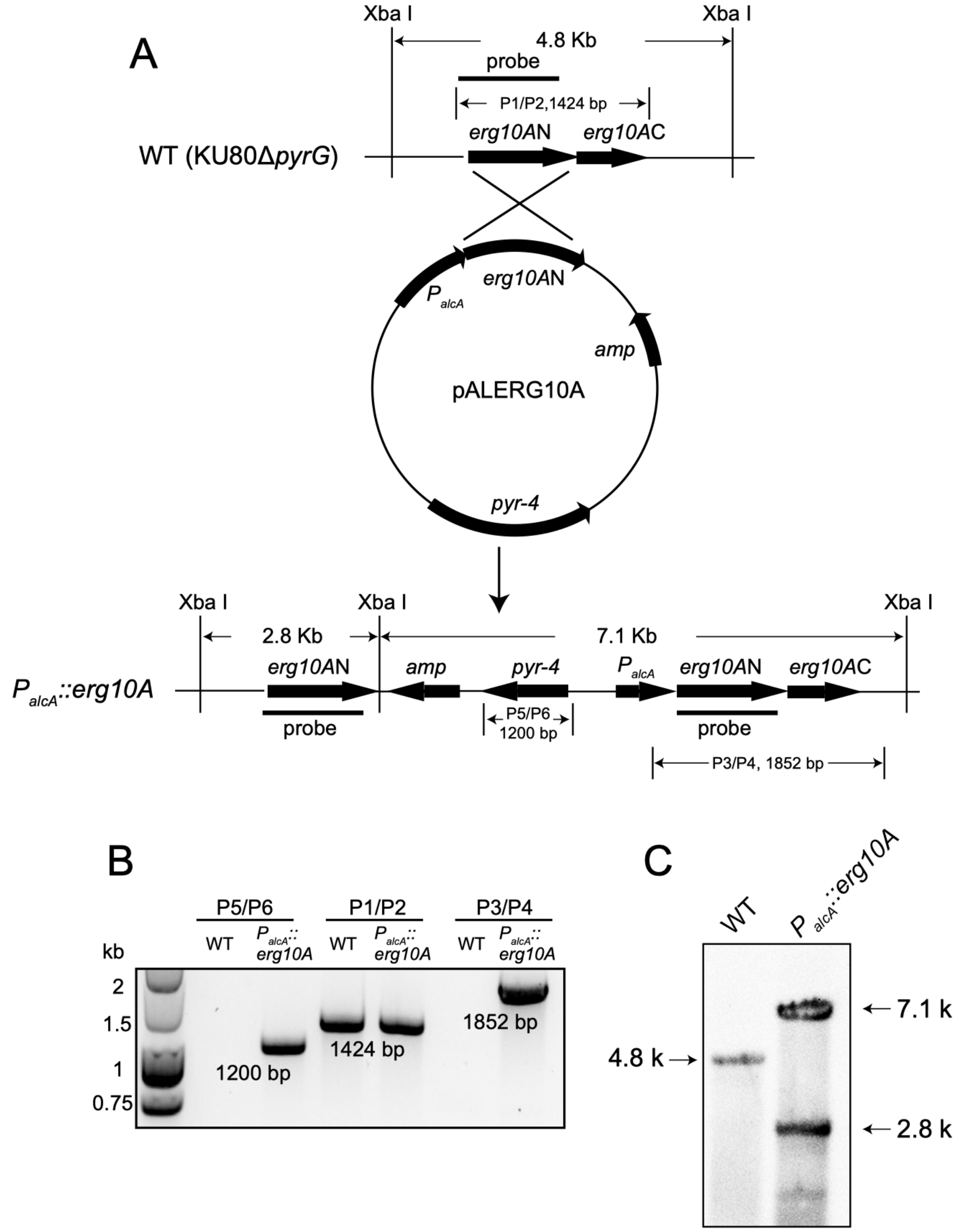

Figure S2. Generation of the P_{alcA}*::erg10A* conditional mutant.

A. Diagram illustrating the replacement of *Aferg10A* native promoter with *A. nidulans alc(A)* promoter.

B. Confirmation of the P_{alcA}*::erg10A* conditional mutant by PCR using primers P1/P2, P3/P4 and P5/P6 as shown in the figure.

C. Southern blot analysis using total genomic DNA digested with *Xba*I and probed by an 800 bp fragment of *erg10A* gene.

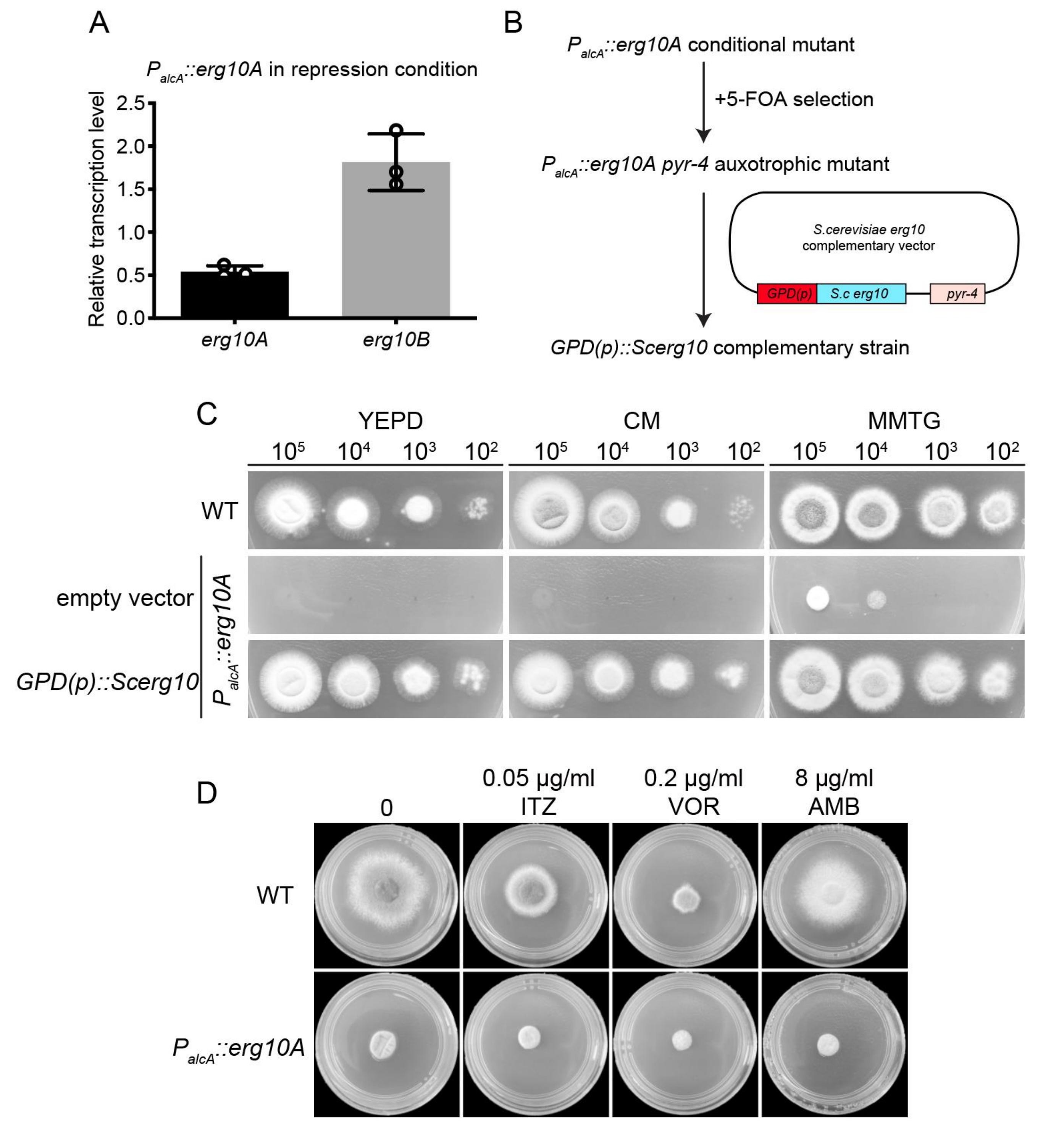

Figure S3. Relative expression level of *Aferg10A* and *Aferg10B*, *S. cerevisiae erg10* complementation in the P_{alcA}::*erg10A* mutant, and sensitivity towards azoles and polyene.

A. The mRNA expression level of *Aferg10A* and *Aferg10B* in the P_{alcA}::*erg10A* conditional mutant under partial repression (MMTG) condition. Gene expression level were normalized to the reference gene *tbp.* Error bars indicate mean ± SD from three independent experiments.

B. Diagram illustrating the complementation of the P_{alcA}::*erg10A* mutant by *S. cerevisiae erg10* gene.

C. Colony morphologies of the wild type and the P_{alcA}::*erg10A* conditional strains overexpressing *Scerg10* gene under the control of GPD constitutive promoter grown on YEPD, CM and MMTG plates for 2 days at 37 °C.

D. 1 x 10^6 conidia of each indicated strain were inoculated on partial repression (MMTG) media supplemented with 0.05 µg/ml itraconazole (ITZ), 0.2 µg/ml voriconazole (VOR) and 8 µg/ml amphotericin B (AMB) and grown for 2.5 days at 37 °C.

Table S1. Data collection and structure refinement

	*Af*ERG10A apo-	*Af*ERG10A-CoA
**Resolution**	48.84 - 2.41	49.62 - 2.44
**Space group**	$P\ 2_12_12_1$	$P\ 2_12_12_1$
**Unit cell**		
***a* (Å)**	61.5	63.3
***b* (Å)**	174.8	173.6
***c* (Å)**	179.1	180.3
**No. of reflections**	297832	268820
**No. of unique reflections**	73595	74187
**$I/\sigma(I)$**	13.7	12.9
**Completeness (%)**	97.0	99.2
**Multiplicity**	3.93	3.59
**R_{merge} (%)**	5.3	7.2
**RMSD from ideal geometry**		
**Bonds (Å)**	0.015	0.014
**Angles (°)**	2.03	1.74
**R_{work} (%)**	20.6	20.5
**R_{free} (%)**	23.6	23.7
**No. of residues**	1588	1596
**No. of water mol.**	197	254
**B factors (Å^2)**		
**Overall**	67.3	56.9
**Protein**	67.5	56.6
**Ligand**	85.7	96.0
**Solvent**	55.5	43.0
**PDB entry**	6L2G	6L2C

Supplementary Reference:

1. Liu W, Xie Y, Ma J, Luo X, Nie P, Zuo Z, Lahrmann U, Zhao Q, Zheng Y, Zhao Y, Xue Y, Ren J. 2015. IBS: an illustrator for the presentation and visualization of biological sequences. Bioinformatics 31:3359-61.
